# High sensitivity C-reactive protein and risk of migraine in a 11-year follow-up with data from the Nord-Trøndelag health surveys 2006–2008 and 2017–2019

**DOI:** 10.1186/s10194-020-01142-1

**Published:** 2020-06-05

**Authors:** Knut Hagen, Lars Jacob Stovner, John-Anker Zwart

**Affiliations:** 1 Department of Neuromedicine and Movement Science, Faculty of medicine and health sciences, Norwegian University of Science and Technology, 7489 Trondheim, Norway; 2 Clinical Research Unit Central Norway, St. Olavs University Hospital, Trondheim, Norway; 3 Norwegian Advisory Unit on Headaches, St. Olavs University Hospital, Trondheim, Norway; 4 Department of Research and Innovation, Division of Clinical Neuroscience, Oslo University Hospital, Oslo, Norway; 5 Institute of Clinical Medicine, University of Oslo, Oslo, Norway

**Keywords:** Migraine, Epidemiology, General population, Follow-up, Inflammation

## Abstract

**Background:**

Several previous studies have reported a cross-sectional association between elevated high sensitivity C-reactive protein (hs-CRP) and migraine. The aim of this population-based follow-up study was to investigate the influence of hs-CRP at baseline on the risk of developing migraine 11 years later.

**Methods:**

Data from the Nord-Trøndelag Health Study performed in 2006–2008 (baseline) and 2017–2019 were used. A total of 19,574 participants without migraine at baseline were divided into three groups based on hs-CRP levels (< 3 mg/L, 3–9.99 mg/L and 10.00–20 mg/L). Poisson regression was used to evaluate the associations between hs-CRP levels and risk ratios (RRs) of migraine, and precision of the estimates was assessed by 95% confidence interval (CIs).

**Results:**

In the multi-adjusted model, increased risk of migraine (RR 1.46, 95% CI 1.05–2.04) was found in the highest hs-CRP levels group compared to the lowest group. In the group with the highest hs-CRP levels, a nearly three times higher risk of chronic migraine (RR 2.81, 95% CI 1.12–7.06**)** was found**,** whereas no evident relationship was found between high hs-CRP level and risk of developing episodic migraine.

**Conclusions:**

The main finding in this 11-year follow-up was that hs-CRP levels between 10.00–20.00 mg/L at baseline was associated with increased risk of chronic migraine.

## Introduction

The pathophysiology of migraine is complex and not fully understood [1]. In the transition from episodic to chronic migraine, several mechanisms have been suggested to be involved, including a sterile inflammation [1]. A non-specific marker of inflammation is high sensitivity C-reactive protein (hs-CRP).

A review published in 2014 of previous cross-sectional studies evaluating the association between hs-CRP and migraine concluded that most studies have found increased hs-CRP levels [2]. This was confirmed in two more recent Norwegian large-scale population-based studies [3, 4]. However, a potential causal relationship between hs-CRP and migraine cannot be evaluated in cross-sectional studies. For this, we need longitudinal studies, and to the best of our knowledge, no previous large-scale population-based follow-up studies have analyzed this.

In the present study, we evaluated the influence of hs-CRP on the risk of developing migraine in a 11-year follow-up. Based on previous knowledge, we hypothesized that elevated hs-CRP increased the risk of migraine.

## Methods

### Study design

This is a population-based historical cohort study. The influence of hs-CRP at baseline was evaluated on the risk of migraine 11 years later**.**

### The HUNT surveys

The present study included data from the two last HUNT surveys conducted in Nord-Trøndelag County, Norway, in 2006–2008 (HUNT3) [5], and in 2017–2019 (HUNT4) [6]. The entire population of the Nord-Trøndelag County aged 20 years of age or more was invited to answer many health-related items in two different questionnaires (Q1 and Q2), including questions about headache, and also a clinical examination, including measurement of weight, height and blood pressure, and blood samples were also taken [5, 6].

### C-reactive protein

**Fig. 1:**
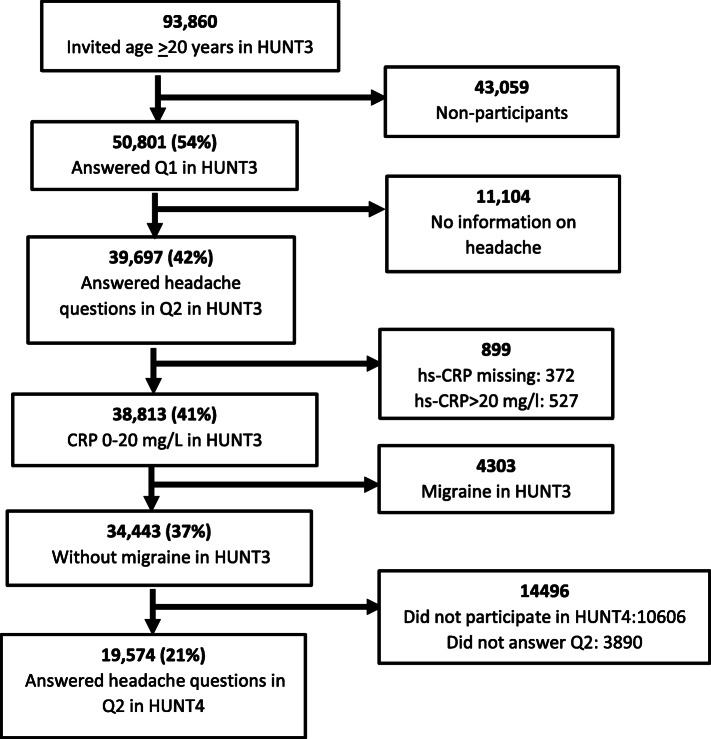
The flow of participants in the present study

Analyses of the blood samples for hs-CRP were done in almost all HUNT3 participants. The method has been described previously [4]. In brief, hs-CRP was analyzed at the Central Laboratory, Levanger Hospital, using Architect cSystem ci8200, by latex immunoassay method. The detection limit was 0.03 mg/L, and samples without detectable hs-CRP were assigned this value. In the present study we divided the participants into three categories based on hs-CRP levels; normal (hs-CRP as 0–2.99 mg/L), medium elevated (3.00–9.99 mg/L), and high (10.00–20.00 mg/L). Participants with hs-CRP values > 20.00 mg/L at baseline, which probably indicate some acute or chronic disease [7], were excluded (*n* = 527) (Fig. 1).

### Headache diagnoses

HUNT3 and HUNT4 had 14 identical headache questions [6]. The initial screening question was “Have you suffered from headache during the last 12 months?”, and those who responded “yes” answered 13 additional questions. A slight modification of the criteria of the International Classification of Headache Disorders, third edition (ICHD 3) [8] was used for diagnosing migraine [9]. As to the modifications of the criteria, individuals would fulfill the migraine criteria even if the attack lasted less than 4 h, because they were not asked for untreated attacks in the question” How long does the headache usually last?” Migraine with aura was diagnosed in those who fulfilled the migraine criteria and answered “yes” to the question “Are the headaches usually characterized by or accompanied by visual disturbance before or during onset (zigzag lines, flickering/flashing light, blurred vision). Chronic migraine was diagnosed in those who fulfilled the criteria of migraine and reported headache ≥15 days/month [6].

### Validity of headache diagnosis

We have previously reported the validity of the questionnaire-based headache diagnoses in HUNT3 [9], and HUNT4 [10]. Merged data of HUNT3 (*n* = 293) and HUNT4 (*n* = 232) gave the following results: for migraine, the sensitivity was 54% and specificity 95% (kappa value 0.52, 95% CI 0.47–0.57) and for migraine with aura, the sensitivity was 39% and specificity 95% (kappa value 0.34, 95% CI 0.30–0.38) [6]. For chronic headache (headache ≥15 days/month, included chronic migraine, chronic tension-type headache and medication overuse headache), the sensitivity was 58% and specificity 99% (kappa value 0.62, 95% CI 0.50–0.74).

### Study population

In HUNT3, 50,803 persons (54%) participated out of 93,860 invited (Fig. 1). Among these, 39,697 (42% of all invited) answered the headache questions, and 38,813 (41%) had measured hs-CRP and had values between 0 and 20.00 mg/l. Among these, 4303 (11.1%) with migraine in HUNT3 were eliminated. Thus, 34, 443 (37%) were without migraine in HUNT3 and constituted the population at risk of developing migraine in HUNT4. A total of 19,574 persons (11,018 women and 8559 men) participated in both HUNT3 and HUNT4 and had answered the headache questions in HUNT4 (Fig. 1).

### Potential confounders

The selection of potential confounders was based on previous literature [11–13]. We evaluated the following potential confounders: Age (10-years categories), gender; duration of education (≤9, 10–12, and ≥ 13 years), body mass index (BMI) (< 25, 25.0–29.9, and ≥ 30 kg/m^2^); smoking (current, previous, and never); alcohol consumption during the last year (never, < 2 times/week, ≥2times/week); total Hospital Anxiety and Depression Scale (0–16, ≥17), headache suffering (yes/no); self-reported diabetes (yes/no); self-reported stroke (yes/no); and self-reported hypertension (yes/no).

### Ethics

This study was approved by the Regional Committee for Medical and Health Research Ethics, the Faculty of medicine, mailbox 8905, 7491 Trondheim. The approval number was #2018/2422/Rek Midt. The participants have given written informed consent.

#### Statistical analysis

A modified Poisson regression with a robust error variance was used to estimate the association between hs-CRP at baseline and risk ratios (RRs) of migraine. Precision of RRs were assessed by 95% confidence intervals (CIs). We present results for three different statistical models separated by number of confounders included; model 1 (age and sex), model 2 (age, sex and BMI), and model 3 (multi-adjusted model testing for all other factors). In model 3 we excluded factors if they did not change RR when evaluating each factor separately or when including several factors grouped together (i.e. self-reported diabetes, stroke, and hypertension). Subjects with missing data on confounding factors (numbers reported in Table 1) were included in the analysis to reduce the impact of possible bias. Analyses were performed with the IBM SPSS version 26 (SPSS, Chicago, Illinois, USA).

**Table 1:** Characteristics of participants (*n* = 19,574) at baseline in HUNT3 related to categories of high sensitivity C-reactive protein (hs-CRP)

	Hs- CRP categories		
	< 3 mg/L	3.0–9.99	10–20 mg/L
Participants, n	15,841	3253	480
Women (%)	54.8	62.2	63.8
Mean age, years (SD)	52.8 (13)	54.6 (14)	53.9 (15)
≥13 years of education (%)	38.0	31.3	31.1
Current smoking, n (%) (missing = 416)	12.5	18.4	18.5
Mean BMI, kg/m^2^ (SD) (missing = 23)	26.6 (4.2)	29.3 (4.9)	29.4 (5.5)
Total HADS score (missing = 184)	6.8 (3.8)	7.1 (5.4)	7.0 (5.2)
Alcohol abstainers during last year (%) (missing = 341)	6.3	7.6	8.9
Self-reported hypertension, n (%)	17.8	27.2	29.4
Self-reported stroke (%)	1.7	2.3	2.3
Self-reported diabetes mellitus, n (%)	3.4	5.5	6.2
Headache (%)	29.3	31.0	31.5

*HADS* ospital Anxiety and Depression Scale, *BMI* body mass index

## Results

Baseline characteristics of the population at risk related to hs-CRP categories in HUNT3 are given in Table 1. As demonstrated, the large group of individuals (*n* = 15,841) with hs-CRP levels < 3.00 mg/l were younger and more likely to have education ≥13 years, and less likely to report diseases or complaints than those with hs-CRP levels ≥3.00.

### Development of migraine at follow-up

In the population at risk (*n* = 19,574), 766 (3.9%) fulfilled the criteria of migraine at follow-up in HUNT4, giving an incidence of 3.6 per 1000 person-years. A total of 354 (1.8%) had migraine with aura, 412 (2.1%) migraine without aura, 711 (3.6%) had episodic migraine) and 55 (0.3%) chronic migraine.

### Risk of migraine

In the analyses adjusting for age and gender, participants with hs-CRP levels between 10.00–20.00 mg/l had increased risk of developing migraine (RR = 1.53, 95% CI 1.10–2.14), migraine with aura (RR = 1.67, 05% CI 1.01–2.76), and chronic migraine (RR = 3.43, 95% CI 1.38–8.52) (Table 2). The multi-adjusted analyses slightly modified the risk ratio, being 1.46 (95% CI 1.05–2.04) for migraine and 1.57 (95% CI 0.95–2.59) for migraine with aura (Table 2). Participants with highest hs-CRP levels (10.00–20.00 mg/l) at baseline had nearly three times increased risk (RR = 2.81, 95% CI 1.12–7.06) of developing chronic migraine in HUNT4 compared to persons with normal hs-CRP (Table 2). For episodic migraine, no evident increased risk was found (RR 1.35, 95% CI 0.94–1.94). No relationship was found between hs-CRP level between 3.00–9.99 mg/l and risk of migraine (Table 2).

**Table 2:** Risk of migraine in HUNT4 based on categories of high sensitivity C-reactive protein (hs-CRP) in HUNT3 evaluated by poisson regression with 95% confidence interval

Hs-CRP (mg/L)	Number	Migraine (overall)	Migraine with aura	Migraine without aura	Episodic migraine	Chronic migraine
		RR (95% CI)	RR (95% CI)	RR (95% CI)	RR (95% CI)	RR (95% CI)
**Model 1** (Adjusted for age and gender)						
< 3.0	15,841	1.00	1.00	1.00	1.00	1.00
3.00–9.99	3253	1.03 (0.86–1.24)	1.00 (0.76–1.33)	1.06 (0.82–1.36)	1.05 (0.87–1.28)	0.76 (0.35–1.66**)**
10.00–20.00	480	1.53 (1.10–2.14)	1.67 (1.01–2.76)	1.50 (0.93–2.42)	1.41 (0.98–2.04)	3.43 (1.38–8.52)
**Model 2** (Adjusted for age, gender and body mass index)						
≤ 2.99	15,841	1.00	1.00	1.00	1.00	1.00
3.00–9.99	3253	0.98 (0.81–1.18)	0.96 (0.72–1.29)	1.00 (0.77–1.29)	1.00 (0.82–1.22)	0.71 (0.33–1.54**)**
10.00–20.00	480	1.44 (1.03–2.03)	1.60 (0.95–2.66)	1.38 (0.86–2.24)	1.32 (0.91–1.92)	3.36 (1.29–8.28)
**Model 3** (Multi-adjusted model^a^)						
≤ 2.99	15,841	1.00	1.00	1.00	1.00	1.00
3.00–9.99	3253	1.00 (0.74–1.35)	0.96 (0.73–1.27)	1.02 (0.80–1.30)	1.01 (0.84–1.21)	0.71 (0.32–1.56**)**
10.00–20.00	480	1.46 (1.05–2.04)	1.57 (0.95–2.59)	1.44 (0.90–2.32)	1.35 (0.94–1.94)	2.81 (1.12–7.06**)**

^a^Multi-adjusted model: Adjusted for age, sex, body mass index, smoking, education level, alcohol use, headache status and Hospital Anxiety and Depression Scale score

## Discussion

In this population-based 11-year follow-up study the main finding was an increased risk of chronic migraine in participants with high hs-CRP levels (10.00–20.00 mg/l) at baseline.

### Comparison with other studies

Most previous migraine studies focusing on hs-CRP have been clinical-based with relatively low number of participants [7]. For example, no association between CRP and migraine was found in a case-control study of 59 participants [14]. Furthermore, no relationship between hs-CRP and headache frequency was reported in a case-control study including 216 migraineurs and 216 controls [15]. Among more recently performed cross-sectional large-scale population-based studies, our main results are in accordance with the results from Tromsø in Northern Norway, including 20,486 participants, reporting an association between elevated hs-CRP for migraine ≥7 days/month (OR 1.22, 95% CI 1.01–1.46), but not for migraine less than 7 days/month [3]. Furthermore, in cross-sectional data from HUNT3 including 38,807 participants, we found the strongest association between elevated hs-CRP for chronic migraine (OR 1.62, 95% CI 1.21–2.17), but no clear association for migraine less than 7 days/month [4].

During the 11-year follow-up, the incidence of migraine was 3.6 per 1000 person-year. In comparison, a much higher incidence of migraine (8.1 per 1000 person-years) was found in a 12-years’ follow-up in Denmark [16]. The reason for the much lower incidence of migraine in the present study is unclear. However, it may in part be explained by the difference in age of population at risk, being 25–64 years in the Danish study and 20–90 years in the present study, and partly because the prevalence of migraine in Denmark increased from 11% to 15% from 1989 to 2001 [17], whereas the corresponding prevalence decreased from 12.0% to 11.1% from HUNT3 to HUNT4 [6].

### Interpretation

The main result in the present 11-year follow-up was that high hs-CRP was associated with increased risk of chronic migraine. This is of relevance in the question of inflammation as an involved mechanism in the transitation from episodic to chronic migraine [1]. One may suggest that an inflammatory response, for which elevated hs-CRP is a marker, may have an impact on peripheral and/or central sensitization, which also may be important mechanisms in the transition from episodic to chronic migraine [1]. Accordingly, it has previously been demonstrated that hs-CRP is related to general pain sensitivity [18, 19].

### Strengths and limitations of the study

The major strengths of this study are the longitudinal population-based cohort design with many participants, a wide age range, and the use of validated diagnoses of headache [10, 11]. In the multivariate analyses, many potential confounding factors were available. However, to avoid over-adjustment bias [20], we adjusted only for the most established confounding factors in epidemiological studies. However, the possibility of residual confounding by an unrecognized factor cannot be ruled out.

Several study limitations should also be considered. Firstly, we have no information about potential risk factors of migraine that may have appeared during the follow-up period before HUNT4. Secondly, generalization of the results to the entire population must be made with some caution, since only 57% in the population at risk in HUNT3 were included in the present study (21% of the invited population in HUNT3), and we cannot be certain that loss to follow-up was random. Thirdly, although the validity of headache ≥15 days/month was good, it should be highlighted that only one person had chronic migraine among participants in the validation studies [9, 10). Thus, the validity of the questionnaire-based diagnosis of chronic migraine could not be reported. Finally, very few (55 participants) of the population at risk developed chronic migraine in HUNT4, giving a wide confidence interval of the estimated risk ratio. On the other hand, it should be highlighted that consistent results have been shown in two large cross-sectional studies [3, 4].

## Conclusions

In this population-based cohort study participants with high hs-CRP levels at baseline had an increased risk of chronic migraine.

## Data Availability

Part of the dataset supporting the conclusions of this article is available on request to the corresponding author. Some of the data are the property of HUNT Research Centre and can only be accessed through direct contact with the Research Centre.
